# Exercise Prescription: Pioneering the “Third Pole” for Clinical Health Management

**DOI:** 10.34133/research.0284

**Published:** 2023-11-28

**Authors:** Zhiwen Luo, Ting Zhang, Shiyi Chen

**Affiliations:** ^1^ Department of Sports Medicine, Huashan Hospital, Fudan University, Shanghai 200040, China.; ^2^ Department of Integrative Medicine, Huashan Hospital, Fudan University, Shanghai, China.

Originating from Western medicine, the concept of “exercise prescription” has gradually gained recognition and promotion in China. Analogous to the traditional medicinal prescription, exercise prescription offers scientific, individualized exercise recommendations to those willing or needing to use exercise as a means of treating ailments, preventing diseases, or enhancing physical constitution [1]. This advice is prescribed formally, detailing elements such as the type of exercise, frequency, intensity, duration, and precautions. Its objectives are straightforward: to treat existing illnesses and to prevent potential ones. Physicians specializing in sports medicine and qualified sports rehabilitation therapists can prescribe such exercise directives, ensuring that recipients get the most appropriate exercise guidance. This prescription is suitable for a diverse demographic, from teenagers to the elderly, and from the health-conscious to those suffering from various chronic diseases.

Exercise, a natural and side-effect-free therapeutic method, has been corroborated by numerous studies for its important efficacy in both disease prevention and treatment, especially for ailments like hypertension, coronary heart disease, stroke, pulmonary diseases, cancer, sports injuries, osteoarthritis, and sarcopenia in the elderly, among at least 27 chronic diseases [2]. For instance, a study encompassing 100,000 samples revealed that prolonged sedentary behaviors coupled with a lack of exercise could amplify the risk of cardiovascular diseases. In contrast, engaging in half an hour of moderate-intensity exercise daily can decrease the risk by 2% [3]. In addition, epidemiological studies indicate that by adhering to 150 min of moderate-intensity exercise weekly, the incidence rate of diabetes decreases by 26%. Regular long-term exercise can reduce the risk of type 2 diabetes by 41.1% and cardiovascular diseases by 33% [4]. In clinical treatments, physical activity has been proven to lower the incidence rates of diabetes and cardiovascular diseases, reduce hospitalization duration, enhance therapeutic outcomes, and cut down on medical costs, bearing significant clinical and societal implications [2]. Alzheimer’s disease, a progressive brain disorder that leads to a gradual loss of memory/thinking/language skills, was reported to be considerably prevented by long-term regular exercise [5,6]. Recently, an economic cost analysis in the UK on exercise intervention for patients with chronic obstructive pulmonary disease showcased that regular physical activity could save patients up to £200 monthly by reducing mortality [7] and hospitalization rates, while exercise was reported to reduce the health care costs by 9.0% to 26.6% around the world [8]. In the context of cancer treatment, our team recently revealed that exercise can metamorphose the immunological microenvironment of non-small-cell lung cancer, transitioning it from “cold” to “hot”, which suggested that exercise increases the number of immune cells such as CD8^+^ T cells, M1 macrophages, etc. and decreases the number of immunosuppressed cells [9]. This provides patients with cancer with a novel, nonpharmacological therapeutic approach.

In addition, the impact of aging on the overall health and quality of life of modern individuals cannot be overlooked. It is reported that approximately 25% of the elderly population struggles with routine activities [10]. Beyond its role in the prevention and treatment of chronic diseases, exercise has been demonstrated to slow the aging process. A systematic review highlighted that, following progressive resistance training interventions for elderly individuals in nursing homes (with an average age range of 80 to 89 years), there were significant improvements in muscle strength outcomes and functional performance measures [11]. In addition, research indicates that moderate-intensity, structured exercise can reduce the risk of mobility disability by at least 18% over a span of 3 years in elderly individuals (1,635 male and female volunteers aged between 70 and 89 years who led sedentary lifestyles) [12]. An increasing body of evidence suggests that exercise emerges as the “third pole” (which means that sports medicine occupies a unique and indispensable position in clinical practice and it is as important as medication and surgery) of clinical treatment, subsequent to medication and surgery. Broadly speaking, whether from the perspective of traditional medicinal theories or contemporary medical research, the irreplaceable role of exercise in preventing and treating a multitude of diseases or delaying aging has been revalidated. In the future, as research delves deeper, our understanding and application of exercise will become more profound, establishing exercise prescriptions as integral components of clinical treatment.

As the general populace grows increasingly health-conscious, the Chinese government has reciprocated with intensified policy support. Ever since the introduction of the “Healthy China Initiative (2019–2030)”, launching a nationwide fitness movement and using exercise to preemptively combat chronic diseases have been elevated to pivotal positions in national strategy [13]. In this project, it was noted that regular and moderate physical activity can help prevent and improve overweight and obesity and chronic diseases such as high blood pressure, heart disease, stroke, and diabetes, as well as promote mental health, quality of life, and well-being, and social harmony. President Xi Jinping has also explicitly emphasized the importance of “promoting health through exercise”, spearheading a new collaborative model among sports, health, and other sectors. These efforts furnish a robust policy framework bolstering the propagation of exercise prescriptions in China.

In the future, the promotion and implementation of exercise prescriptions need to be more refined and systematic. Beyond a simple exercise suggestion, it represents a scientific, holistic health management plan. Moreover, the integration with traditional medicine, such as the combination of exercise with medications or physiotherapy, will be a significant avenue for future developments. At the same time, establishing a nationwide database for exercise prescriptions to realize data sharing and cross-regional services is also the trend. The future of exercise prescriptions in China is rapidly evolving. With the backing of national health policies and an ever-growing health consciousness among the population, exercise prescriptions will encounter unprecedented opportunities. Below are its new development directions:

1. Customized and personalized prescription services. Tailoring exercise plans according to individual circumstances, such as age, gender, basic health condition, and physique. In addition, there is a need to consider the combination of various chronic diseases and exercise prescriptions to guarantee optimal therapeutic results.
2. Integration of exercise prescriptions with modern medical equipment. By harnessing advanced medical technology, like wearable devices and artificial intelligence, patients’ exercise data can be monitored in real time, offering immediate feedback and suggestions [14]. This not only ensures the efficacy of the exercise but also adeptly sidesteps potential injuries resulting from inappropriate exercise.
3. Training and professional talent pool. Given the specialization of exercise prescriptions, specialized training and talent reserves are imperative. Multiple hospitals have already inaugurated specialized outpatient clinics for exercise prescriptions. Still, a more widespread promotion requires a greater number of professionals, making intensified education and training in medical institutions crucial.
4. Widespread popularization and publicity. Although many recognize the significance of exercise, comprehension of exercise prescriptions remains shallow. Both government and healthcare institutions at all levels should intensify popularization and publicity efforts for exercise prescriptions, truly integrating it into everyone’s daily life. Such efforts not only reduce the incidence of chronic diseases and decrease financial expenditures for the health sector but also enhance the quality of life and life expectancy of the populace.
5. Establishment of comprehensive assessment and feedback mechanisms. To assure the efficacy and safety of exercise prescriptions, a thorough evaluation and feedback mechanism is mandatory. This includes, but is not limited to, routine physical examinations, analysis of exercise data, and individualized adjustment recommendations.
6. Integration of clinical and foundational research. Numerous randomized controlled trials have proven the efficacy of exercise prescriptions in treating various chronic diseases, such as cardiovascular diseases, diabetes, and cancer. Concurrently, foundational research has elucidated the benefits of exercise at the molecular, cellular, and tissue levels, such as modulating immune responses, cellular autophagy, and cardiovascular health. A model from basic research to clinical translational research could facilitate the widespread use of exercise prescriptions in the clinic [15].

From the ancient practice of “Xiong Jing Niao Shen” to the modern “exercise prescription”, the concept of exercise as a health and therapeutic method has been deeply ingrained. Today, exercise prescriptions, as the perfect amalgamation of medicine and sports, are gradually being popularized and applied. In the future, with technological advancements and societal progress, exercise prescriptions will be more refined and individualized, providing a more scientific and rational health management service to the public. Although China’s experience may be difficult to apply directly to a global setting due to differences in policy and national conditions, such medical development experience will certainly provide some insight to other countries. Building on this foundation, it is anticipated that China’s sports medicine research will cooperate with other countries, contributing to the realization of a healthy and joyous life for all citizens.
